# Nanoparticles of *N*-Vinylpyrrolidone Amphiphilic Copolymers and Pheophorbide *a* as Promising Photosensitizers for Photodynamic Therapy: Design, Properties and In Vitro Phototoxic Activity

**DOI:** 10.3390/pharmaceutics15010273

**Published:** 2023-01-12

**Authors:** Alexander Yu. Rybkin, Svetlana V. Kurmaz, Elizaveta A. Urakova, Natalia V. Filatova, Lev R. Sizov, Alexey V. Kozlov, Mikhail O. Koifman, Nikolai S. Goryachev

**Affiliations:** 1 Federal Research Center of Problems of Chemical Physics and Medicinal Chemistry, Russian Academy of Sciences, Pr. Akademika Semenova 1, 142432 Chernogolovka, Russia; 2 Faculty of Fundamental Physical and Chemical Engineering, Lomonosov Moscow State University, 119991 Moscow, Russia; 3 Department of Chemistry and Technology of Macromolecular Compounds, Ivanovo State University of Chemistry and Technology, Sheremetevskiy Av. 7, 153000 Ivanovo, Russia

**Keywords:** nanoparticles, *N*-vinylpyrrolidone, (di)methacrylates, amphiphilic copolymers, pheophorbide, chlorin e_6_, PDT, photodynamic action, photosensitizer

## Abstract

A series of nanoparticles (NPs) with a hydrodynamic radius from 20 to 100 nm in PBS was developed over the solubilization of hydrophobic dye methyl pheophorbide *a* (chlorin e_6_ derivative) by amphiphilic copolymers of N-vinylpyrrolidone with (di)methacrylates. Photophysical properties and biological activity of the NPs aqueous solution were studied. It was found that the dye encapsulated in the copolymers is in an aggregated state. However, its aggregation degree decreases sharply, and singlet oxygen quantum yield and the fluorescence signal increase upon the interaction of these NPs with model biological membranes—liposomes or components of a tissue homogenate. The phototoxic effect of NPs in HeLa cells exceeds by 1.5–2 times that of the reference dye chlorin e_6_ trisodium salt—one of the most effective photosensitizers used in clinical practice. It could be explained by the effective release of the hydrophobic photosensitizer from the NPs into biological structures. The demonstrated approach can be used not only for the encapsulation of hydrophobic photosensitizers for PDT but also for other drugs, and *N*-vinylpyrrolidone amphiphilic copolymers show promising potential as a modern platform for the design of targeted delivery vehicles.

## 1. Introduction

Insufficient effectiveness of current cancer treatment methods is a major problem at present, which is urging the development and improvement of new non-invasive treatment methods. One such promising method is photodynamic therapy (PDT). This method combines the double selectivity of action, which is achieved by the administration of a special drug, a photosensitizer (PS), which is non-toxic in the dark and can selectively accumulate in the tumor, followed by the local irradiation of the tumor with the red or near IR light [1,2,3]. PDT has been used in clinical practice for over 30 years and has demonstrated promising results. PDT has several advantages: It is non-invasive, painlessness, no side effects on the healthy tissues and organs, has the possibility of combination with other treatment methods, and it also could enhance the immune response against cancer cells [1,2,3,4]. However, the development of PDT is hampered by the drawbacks of PSs, currently used in clinical practice [2,5]; thus, researchers worldwide are looking for ways to create new and more effective PSs [5,6,7,8,9].

The main problem of obtaining an effective PS is a long list of sometimes contradictory requirements of a modern PDT method. According to it, PS should have high absorbance in the red or near IR region of the spectrum in the so-called “tissue transparency window” (1), a high quantum yield of reactive oxygen species (2), be non-toxic in the dark (3), be soluble in water (4), selectively accumulate in the tumor (5), have strong fluorescence signal for diagnostics (6), and others [3,10,11].

Researchers have recently paid close attention to nanoscale systems as a tool to deliver PS [7,12,13,14,15]. The use of such nanoscale systems could provide a number of advantages: significantly increase the selectivity of PS accumulation in tumor cells, increase its stability, and improve solubility in biological media. From this point of view, the use of polymer nanoparticles looks extremely promising [12,13,14].

Nanosized systems of biologically active compounds (BAC) of various natures based on amphiphilic copolymers of *N*-vinylpyrrolidone (VP) with (di)methacrylates can be obtained in a simple way [16]. The method for their preparation includes the BAC encapsulation into nanoparticles of VP amphiphilic copolymers at concentrations close to the critical concentration of aggregation in isopropyl alcohol and subsequent dissolution of polymer films in aqueous media. The drug could associate with the copolymers through its physical entrapment within the polymer matrix, and the drug molecules penetrate into their cavities with the formation of “guest-host” complexes, or the drug is adsorbed on the surface of the nanoparticles. Hydrophobic BACs are located in the internal cavities—the low-polarity core of the polymer particle, and hydrophilic ones—in its polar shell, which consists of VP units. They are retained by the polymer particle due to hydrophobic interactions and/or through the hydrogen bond formed by their functional groups with the C=O groups of the lactam cycle of VP units.

Here, we propose the *N*-vinylpyrrolidone (VP) copolymer and terpolymers branched with triethylene glycol dimethacrylate (TEGDM) as the carrier of hydrophobic PS for PDT. We have previously demonstrated that branched VP copolymers of different compositions, molecular weight, and topology do not significantly affect the viability of Vero cells and tumor cells A-172 and Hela, and such polymers are biocompatible [17,18,19,20]. Furthermore, the organic complex of platinum(IV) [18] and anticancer drug doxorubicin [21] encapsulated in these copolymers have less cytotoxicity than the free ones. The dimensions of macromolecules and their aggregates in aqueous media correspond to the main criteria for delivery vehicles and are related to their excretion from the body by the kidneys and spleen [22,23,24], and small particles will circulate in the bloodstream for a sufficient time to increase the efficiency of the encapsulated drugs.

Thus, the current work aimed to create the original water-soluble nanoscale systems based on polymer nanoparticles and already known PS and study their photophysical properties and photodynamic activity to identify the most promising systems as photosensitizers for application in photodynamic therapy.

## 2. Materials and Methods

### 2.1. Photosensitizers

Methylpheophorbide *a* was obtained by extraction from *Spirulina microalgae* with methanol in the presence of sulfuric acid according to the method [25]. In this method, the extracted chlorophyll(a) demetallization and the simultaneous transesterification of acidic groups occur. The resulting methyl pheophorbide was concentrated using a rotary evaporator and purified by chromatography on silica gel using dichloromethane-diethyl ether as eluent. The MALDI-TOF, UV-Vis, and IR of methylpheophorbide *a* coincide with those given in [26].

**Figure 1:**
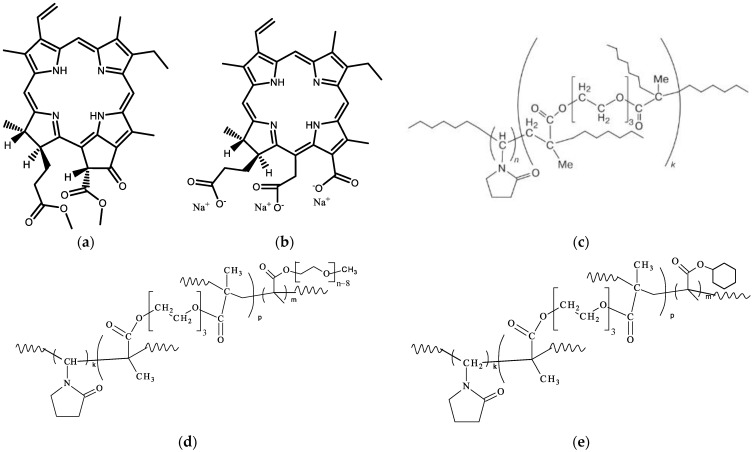
Chemical structure of methylpheophorbide *a*, MPP (**a**), reference PS chlorin e_6_ sodium salt (Ce6), (**b**) and copolymers: VP−TEGDM (CPL1) (**c**), VP−PEGMEM−TEGDM (CPL3, CPL5) (**d**), VP-CHM-TEGDM (CPL12) (**e**).

Reference PS—chlorin e_6_ sodium salt (Ce6, Figure 1b) was synthesized from methylpheophorbide *a* according to the known method [27,28] (Supplementary Figure S1).

### 2.2. Synthesis of Copolymers

The monomer VP (Alfa Aesar) was purified by vacuum distillation to remove the NaOH inhibitor. The monomers TEGDM, PEGMEM, cyclohexyl methacrylate (CHM) (Aldrich) were used without additional purification, isopropyl alcohol (IPA, Khimmed, Russia) of the extra purity grade. The initiator, azobisisobutyronitrile (AIBN), was purified by recrystallization from ethanol.

The copolymers of different monomer compositions, such as VP-TEGDM copolymer (CPL1), VP-PEGMEM-TEGDM copolymer (CPL5), and VP-CHM-TEGDM (CPL12) were obtained by radical copolymerization in toluene at monomer molar ratio of 100:5 and 98:2:5, respectively, without any chain growth regulators. The VP-PEGMEM-TEGDM copolymer (CPL3) was synthesized from the 95:5:5:5 monomer mixture in toluene in the presence of 1-decanethiol (DT) as a chain transfer agent [19]. The copolymers were isolated from the reaction mixture by precipitation with a 10-fold excess of hexane. The obtained copolymers were described in detail elsewhere [17,18,19,29,30].

### 2.3. Encapsulation of Photosensitizers into Nanoparticles

The MPP encapsulation into CPL1 was carried out using a solution of the copolymer with a concentration of 3.7 mg/mL in isopropyl alcohol (135 mL) and a solution of MPP (0.7 mg/mL) in toluene (4.2 mL). The MPP encapsulation into CPL3, CPL5, and CPL12 was carried out according to the following procedure: a solution of the copolymer with a concentration of 3.5 mg/mL was prepared in isopropyl alcohol (150 mL). A solution of MPP (0.7 mg/mL) with a volume of 11.2 mL was prepared in toluene.

The resulting copolymer solutions were placed in a flask with a magnetic anchor and put on a magnetic stirrer in the mode of the intensive mixing of the solutions. The dye solution was gradually (one drop at a time) added to the flask with the vigorously stirred copolymer solutions. The resulting solutions were dried to a minimum volume (approximately 2 mL) using a rotary evaporator. The remaining part of the solution was poured into a Petri dish and dried under a draft for several hours and in a vacuum at room temperature. Polymer films containing MPP were ground to powder for convenient storage and further use. The resulting powders of MPP compositions were dissolved in phosphate-buffered saline (PBS, pH 7.2–7.4) or water immediately before the experiment; the dye concentration in the solution (M) was determined by the weight of the sample of dry powder.

In order to evaluate the optimal amount of MPP, which could be encapsulated in copolymer NPs, we did a preliminary test. NPs were prepared with various MPP loadings based on CPL1 and CPL5. We varied the volume of the dye solution in toluene (0.15, 0.3, 0.6, and 0.9 mL) added to the solution of the copolymer in isopropyl alcohol (4 mL, 3.5 mg/mL) and obtained NPs, where MPP content per copolymer was 0.75, 1.5, 3.0, and 4.5%. Absorption spectra of the obtained NPs in PBS showed the linear dependence A_675nm_ ([MPP], wt.%) for the dye content up to 3%. Thus, based on this result, we obtained NPs with the mass fraction of MPP in the powder, which was 0.6 and 1.5% calculated for CPL1 + MPP and CPL3 + MPP, CPL5 + MPP, CPL12 + MPP, respectively.

### 2.4. Analysis of Copolymers

*Elemental Analysis of copolymers.* The content of CHNS atoms in the copolymers was determined by elemental analysis on a CHNS/O instrument Vario MICRO cube (Elementar Analysensysteme GmbH, Langenselbold, Germany, 2007). The nitrogen content in CPL1, CPL3, CPL5, and CPL12 was 10.9, 8.6, 9.5, 10.3%, respectively. The sulfur atom in CPL3 was 0.8%.

*IR Spectroscopy.* The copolymers and their compositions with a dye were identified using IR-spectroscopy. The IR spectra were recorded in ATR (Attenuated Total Reflection) mode, the spectral region was 400–4000 cm^−1^, and the number of scans was 16. The FTIR spectrometer Bruker α was used.

*Size Exclusion Chromatography.* The absolute molecular weights of the copolymers were determined by size exclusion chromatography using a Waters liquid chromatograph ((2 columns PS-gel, 5 μm, MIXED-C, 300 × 7.5 mm) (Waters Corp., Milford, MA, USA)) equipped with a refractive index detector and a multi-angle light scattering detector WYATT DAWN HELEOS II (Wyatt Technology, Santa Barbara, CA, USA), λ = 658 nm. The eluent was *N*-methylpyrrolidone with the addition of lithium chloride (1 wt%), which prevents the aggregation of macromolecules in a polar solvent. The measurement temperature was 70 °C; the elution rate was 1 mL min^−1^. The d*n*/d*c* values were determined from multi-angle light scattering detector data. All copolymer solutions (10–20 mg mL^−1^) were preliminarily filtered through filters with a pore diameter of 0.2 μm. The absolute weight average molecular weight of the copolymers was obtained from light scattering data using the Astra software version 5.3.2.20 (Wyatt Technology).

### 2.5. Dynamic Light-Scattering Experiments

The hydrodynamic radii, *R*_h_, of copolymers and MPP-copolymer structures in PBS were determined by dynamic light scattering (DLS). Before preparing samples for measurements, the solutions were filtered using a PES syringe filter with a pore diameter of 0.45 μm. The vials with the solution were thermostated at a given temperature for 20 min. DLS measurements were carried out using a Photocor Compact instrument (Photocor LTD., Moscow, Russia) equipped with a diode laser operating at a wavelength of 654 nm. Solutions of copolymers and MPP-copolymer structures were analyzed at a detection angle of 90 °C. The experimental data were processed using the DynaLS software, version 2.8.3 (Dr. Alexander A Goldin (Alango Ltd., Haifa, Israel), updated in March 2002). The size distribution curves for scattering centers were obtained by processing the results of measurements of scattering intensity fluctuations by solutions. The hydrodynamic radii *R*_h_ of the MPP-loaded copolymer particles were calculated using the Einstein–Stokes equation

*D* = *kT*/6π*ηR*, (1)

where *D* is the diffusion coefficient, *k* is the Boltzmann constant, *T* in the equation is the absolute temperature (K), and *η* is the viscosity of the medium, in which the dispersed particles are suspended.

### 2.6. Photophysical and Photochemical Studies

Absorption spectra were measured using a Cary-60 spectrophotometer equipped with a thermostated cell. Fluorescence steady-state spectra of the dyes under study were recorded by a Cary-Eclipse fluorescence spectrophotometer.

The photochemical activity of the studied compounds was measured by a setup based on a Specord M-40 spectrophotometer equipped with an interface for computer recording of spectra, a temperature-controlled cell compartment (set to 20 °C) with integrated LEDs, and an Arduino-based control unit. The cuvette with the solution was illuminated by two LEDs (λ = 660 nm); the light irradiance was 5 mW/cm^2^. Each sample was irradiated for 2 s, after which the absorption spectrum was recorded in the range of 350–700 nm. In total, there were 12 irradiation cycles for each sample.

*Detection of singlet oxygen* ^1^O_2_ formation has been performed by a standard method using 1,3-diphenylisobenzofuran (DPBF) [31]. To introduce a hydrophobic probe DPBF to an aqueous solution, it was incorporated into the phosphatidylcholine liposomes using a modified method [32,33]. The aqueous solution containing DPBF (0.05 mM of DPBF in liposomes, 0.5·mM of lecithin) and the photosensitizer (5 μM), in a 10 × 10 mm quartz cuvette was irradiated with the setup, as described above. To maintain a constant oxygen concentration, the cuvette was constantly bubbled with air. The decrease of the DPBF probe absorbance was registered at 420 nm. The experiment was carried out at least three times for each photosensitizer. Relative singlet oxygen quantum yield was calculated by the following method [31] using chlorine e_9_ trisodium salt as a reference (Φ_Δ_ = 0.75 [34]).

### 2.7. Phototoxicity Evaluation on HeLa Cell Line

*Cell culture.* Cell line M-HeLa (human cervical adenocarcinoma cells, M subclone) was used to evaluate the photodynamic activity of the compounds under study. Cells were obtained from the Russian collection of vertebrate cell cultures (Institute of Cytology RAS, St. Petersburg, Russia). Cells were grown in an atmosphere of 5% CO_2_ at 37 °C. DMEM medium without a phenol red dye was used to exclude the possibility of its photodynamic influence on the experimental results, since this dye has high absorption in the red spectral region. The medium was supplied with 10% fetal calf serum (Biowest, Nuaillé, France) and a Penicillin-Streptomycin mixture. Cells were seeded in a 96-well plate at ~5000 cells per well.

*Cell incubation of the compounds under study.* After 24 h of seeding, compounds under study were added to the wells, the pure medium was added to the control wells, and cells were incubated for 24 h. As a positive control, a chlorin e_6_ sodium salt (Ce6) was used. Before irradiation, the medium was replaced with a fresh one (without the compound under study). For comparison, two identical sets of 96-well cell plates were made to estimate light and dark toxicity. The latter set of plates was kept in the dark.

*Irradiation.* Cell plates were illuminated for 30 min with a specially designed illuminator as described in [35]. It was based on a 500 W halogen lamp with a set of cut-off glass filters with λ > 630 nm. An additional scattering thin plastic film filter was used to provide equal light flux for all wells of the plate. A water filter removed heat and IR irradiation from the lamp. The lamp was cooled by an air fan, and the temperature of the 96-well plate holder was controlled by a water thermostat (37 °C). The total radiant dose was 41.4 J/cm^2^ (30 min exposure time with 23 mW/cm^2^ light irradiance).

*MTT method.* After irradiation, cells were incubated for 24 h and stained with MTT (3-(4,5-dimethyl-2-thiazolyl)-2,5-diphenyl-2H-tetrazolium bromide) [36]. MTT (0.5 mg/mL) was added to each well, and the cells were held in an incubator for 3 h, then the cell medium was removed, and the obtained formazan crystals were dissolved in DMSO (0.1 mL/well) for 10 min on a microplate shaker. The optical density of formazan was evaluated at 570 nm with a SPARK 10 M device (Tecan, Männedorf, Switzerland). MTT staining of control cells was taken as 100%. The IC_50_ dose (concentration of a compound that reduces MTT staining by 50%) was found using the median effect analysis [37]. The results are presented as mean ± SD, calculated from at least three independent experiments.

### 2.8. Interaction of Nanoparticles with Liposmes and Tissue Homogenate

*Preparation of liposomes solution.* Phosphatidylcholine (lecithin) from egg yolk (Sigma) was used to prepare liposomes’ solution according to the method described in [32,33]; lipid concentration in the cuvette was 100 μM.

*Preparation of mouse brain homogenate.* All the animal experiments were performed according to compliance with the EU Directive 2010/63/EU for animal experiments and were approved by the Ethical Committee of FRC PCPMC RAS. Hybrid BDF1 mice (about six months old) were sacrificed by decapitation. The freshly prepared brains were thawed and homogenized using a WisdWiseTis HG-15D homogenizer (Daihan Scientific Group, Wonju, Republic of Korea) for 2 min in a buffer (0.1 M Tris-HCl, pH 7.4). Protein concentration was determined by the Lowry method [38]. Obtained homogenate was stored in liquid nitrogen (−196 °C) before use. For fluorescence measurements (Section 3.8), it was diluted with the buffer (0.1 M Tris-HCl, pH 7.4) to achieve a 0.01 mg/mL protein concentration in the cuvette.

## 3. Results and Discussion

### 3.1. The VP Copolymer Parameters, Structure and Properties

The copolymers with chemical structures (Figure 1) have been obtained by radical copolymerization in toluene. Bifunctional and monofunctional methacrylic comonomers such as TEGDM, PEGMEM, and CHM, like MMA [39], were more reactive in radical copolymerization than VP. This conclusion followed from our study of the copolymerization kinetics in a wide range of VPs and dimethacrylate conversions and the analysis of the monomer composition of the resulting copolymers by IR spectroscopy and isothermal calorimetry [40]. The rate of VP conversion is much lower than that of dimethacrylate, and, as a result, all radicals add more active monomers at the initial stage of copolymerization, and polymer chains are formed enriched with (di)methacrylate units. Their distributions in growing polymer chains are statistical, and their bulky substitutions limit intermolecular cross-linking, leading to insoluble macrogel formation. The copolymer structure formed at the initial stages of the reaction contains “pendant” C=C bonds of TEGDM units, through which growing VP chains are attached [40]. The proposed topological structures of the copolymers are shown in the Supplementary Figure S2. The branched nature of these copolymers was demonstrated through the analysis of the dependence of *M*_w_ on the eluent volume *V*_r_ and root-mean-square radii of gyration on *M*_w_ values and comparison with those for linear PVP in [20].

In IR spectra of copolymers, absorption bands at wavenumbers of ~1720 and ~1665–1660 cm^−1^, respectively, corresponding to the stretching vibrations of C=O groups in (di)methacrylate and VP units were observed (Supplementary Figure S3). In the range of 3600–3000 cm^−1^, a broad absorption band is typical of the stretching vibrations assigned to OH groups of adsorbed water linked by hydrogen bonds to amphiphilic VP copolymers [41]. The absorption band at 1100 cm^−1^ corresponding to the vibrations of the ether –C–O– bond in PEGMEM oligomeric block in the IR spectra of CPL3 and CPL5 was observed. The intensity of absorption bands related to stretching vibrations of –CH_2_– groups in the region of 3000–2800 cm^−1^ increases due to the appearance of PEGMEM units and DT residues in CPL3. In these regions of the CPL12 IR spectrum, the intensity of absorption bands related to the stretching and vibration of –CH_2_– groups increases, too, as a result of the presence of CHM units (Supplementary Figure S3d).

Table 1 shows the physicochemical characteristics of these copolymers as molar compositions calculated from the data of elemental analysis, absolute molecular masses (*M*_w_), the critical concentration of aggregation (CAC), and hydrodynamic radii (*R*_h_) in water. The given data shows that (di)methacrylates content in copolymers varies from ~5 to 10 mol% depending on the monomer mixture composition. The sulfur content was also calculated for the CPL3 prepared in the presence of DT (3.6 mol%). Thus, it contains the –SC_10_H_21_ group as the residue of the chain transfer agent and can be toxic to cells (see Section 3.6). The VP copolymers differ markedly in molecular weight.

The VP copolymers consist of hydrophilic VP monomers and hydrophobic comonomers, such as TEGDM, PEGMEM, and CHM, with a cyclic aliphatic substitute. They can be considered micelle-like formations in which polar and low-polarity moieties are formed by VP units and TEGDM, PEGMEM, or CHM units, respectively. In addition, the remains of DT (nonpolar groups—SC_10_H_21_) are built into the polymer radicals with methacrylates ends, and CPL3 becomes the most hydrophobic in a series of prepared copolymers. According to the cryo-TEM image [42], the hydrophobic fullerene C_60_ molecules were visible in the core of micellar particles of VP-TEGDM copolymer obtained in the presence of DT.

In water, these amphiphilic copolymers can exist as monomolecular micelles with a low-polarity nucleus and polar shell and/or their aggregates at the above CAC (Table 1) as result of their self-assembly. The CAC values were determined from the DLS data, namely the dependence of the intensity of light scattering on the concentration of the copolymer in water. The semi-logarithmic dependences of the intensity of light scattering on the concentration of the copolymer were approximated by two linear functions. Their intersection can be evaluated as the copolymer CAC in water. The CAC value of the copolymers depends on their chemical composition and molecular mass. CPL12 containing hydrophobic CHM units are most prone to aggregate formation. The hydrodynamic radius *R*_h_ of light scattering in water was shown at Table 1; the nanosized particles of copolymers were in it. The distribution of intensity in the size of particles in CPL12 water solution, unlike other polymers, is unimodal (Supplementary Figure S4), and the *R*_h_ value in the maximum of the peak is ca. 50 nm, which slightly increases with a temperature growth up to 45 °C. There is a tendency to increase the average intensity of light scattering as a result of the aggregation increasing of temperature-responsive copolymer. Consequently, the CPL12 water solution is opalescent.

### 3.2. The Dye Encapsulation into Polymer Particles

The dye encapsulation into polymer particles was carried out in alcohol solutions as a thermodynamically suitable solvent for amphiphilic copolymers. Moreover, hydrophobic MPP is poorly soluble in isopropyl alcohol, which facilitates its association with the copolymer. Dye association with the copolymer particles can be through the physical entrapment of the drug within the polymer matrix and penetration of the molecules into their cavities with the formation of complexes such as the “guest-host” type. Hydrophobic interactions between dye and (di)methacrylates moieties stimulate the molecules’ penetration into the nucleus of mono- or multimolecular micelles formed as a result of aggregation [43,44]. In a water solution, these interactions are enhanced, and sufficiently stable nanostructures are formed. Photos of dry powders and water solutions of obtained CPL1 + MPP, CPL3 + MPP, CPL5 + MPP, CPL12 + MPP nanoparticles are presented in Supplementary Figure S2c–f. The solutions were clear or slightly opalescent.

We assume that all MPP molecules are encapsulated in the copolymer NPs under study, since the native MPP is completely insoluble in PBS or other water solutions. Furthermore, we did not observe any solid residue on the bottom of all water solutions of MPP NPs, even after weeks of storage of the solutions (Section 3.8).

The IR spectrum of the MPP powder is shown in Supplementary Figure S3. It is difficult to identify the dye in compositions due to its low content in these compositions and its overlapping with the absorption bands of copolymers. However, it is noteworthy that the intensity of the absorption bands of the copolymers changes in MPP presence. The absorption band of the stretching vibrations of C=O group in VP units in the IR spectrum of MPP composition based on CPL1 is shifted from 1658 to 1648 cm^−1^. The absorption band intensities are redistributed in the 1300–1200 cm^−1^ region (Supplementary Figure S3a). The absorption band at a wavenumber of 956 cm^−1^ changes in CPL3-MPP composition (Supplementary Figure S3b). This band is even more intense in MPP compositions based on CPL5 and CPL12; the absorption band at 818 cm^−1^ becomes more pronounced (Supplementary Figure S3c,d).

### 3.3. Studies of Nanoparticles Size by Dynamic Light Scattering

**Figure 2:**
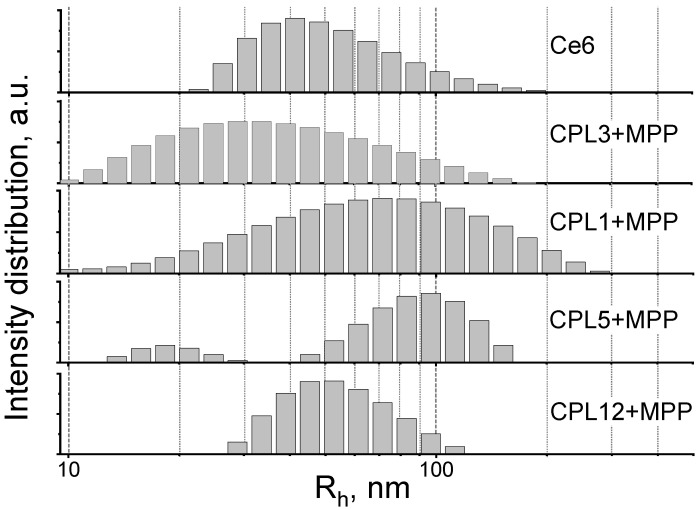
DLS profiles of nanoparticles under study. The PS concentration—10 μM, PBS solution, 20 °C.

In aqueous solutions (PBS), all the obtained [copolymer]-MPP systems form nanoparticles, the average hydrodynamic sizes of which vary from 27 to 95 nm (Figure 2 and Table 2). The reference PS—trisodium salt of chlorine e_6_ (Ce6) also forms aggregates in water (Figure 2 and Table 2), which is in good agreement with the literature data [45,46].

Here, we should note that specific nanoparticle size could significantly improve their accumulation in the cancer tissue due to the so-called “enhanced permeability and retention effect” (EPR) or passive targeting effect, which is based on the defects in the vasculature and other physiological properties of tumors. The optimal size of NPs for passive targeting could vary from the tumor type and is considered to be around 200 nm [47,48].

### 3.4. Absorbance and Fluorescence Spectra of Nanoparticles

**Figure 3:**
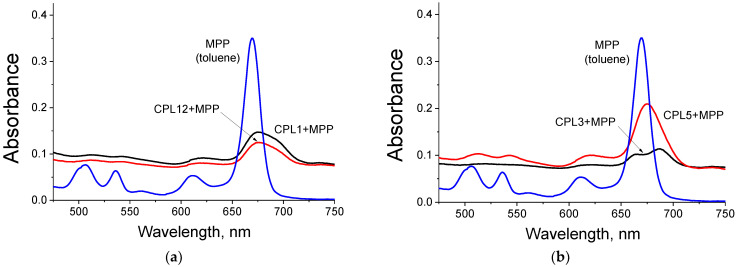

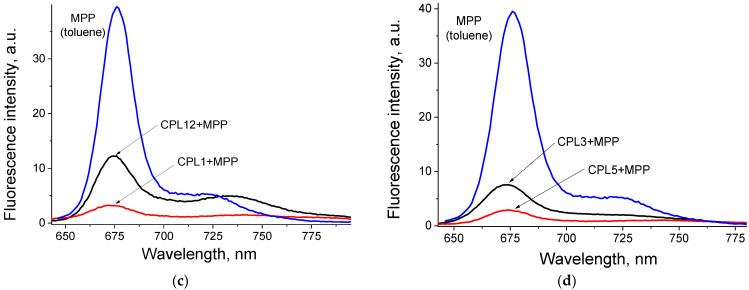
Absorption spectra (**a**,**b**) and fluorescence spectra (**c**,**d**) of nanoparticles in water solution and native MPP in toluene. For all fluorescence spectra λ_ex_ = 420 nm. Concentration of MPP in NPs is 5 μM in water, native MPP in toluene—2 μM.

As can be observed from Figure 3a,b, the spectra of MPP embedded in NPs differ significantly from the spectra of the native dye in toluene. For all obtained NPs, we observe a significant (from 10 to 20 times) decrease in absorption in the region of the Q absorption band compared to the absorption spectrum of MPP in toluene (Figure 3a,b). We also observe a peak broadening, accompanied by a bathochromic shift of 6–18 nm (Figure 3a,b). This picture is quite typical for H-aggregates of porphyrin derivatives. We believe that the formation of MPP aggregates inside the hydrophobic core of copolymer nanoparticles can explain the observed effects.

As can be observed from Figure 3c,d, the fluorescence of MPP embedded in NPs is strongly quenched compared to that for the original dye in organic solvent (from 65 to 258 times, Table 2). These data confirm our assumption that these dyes are located in the hydrophobic core of NPs in the form of aggregates. It is known that H-aggregates of dyes do not have fluorescence. In contrast, the fluorescence of J-aggregates is significantly quenched.

### 3.5. Singlet Oxygen Generation

The generation of ^1^O_2_ upon irradiation of the studied compounds was measured using the hydrophobic probe DPBF embedded in the structure of lecithin liposomes (Materials and methods, Section 2.6). Under these conditions, reference PS Ce6 is able to efficiently generate singlet oxygen when irradiated with red light (λ = 660 nm), which is in good agreement with the literature data. It is known that the value of Φ_Δ_ for Ce6 = 0.75 [34].

**Figure 4:**
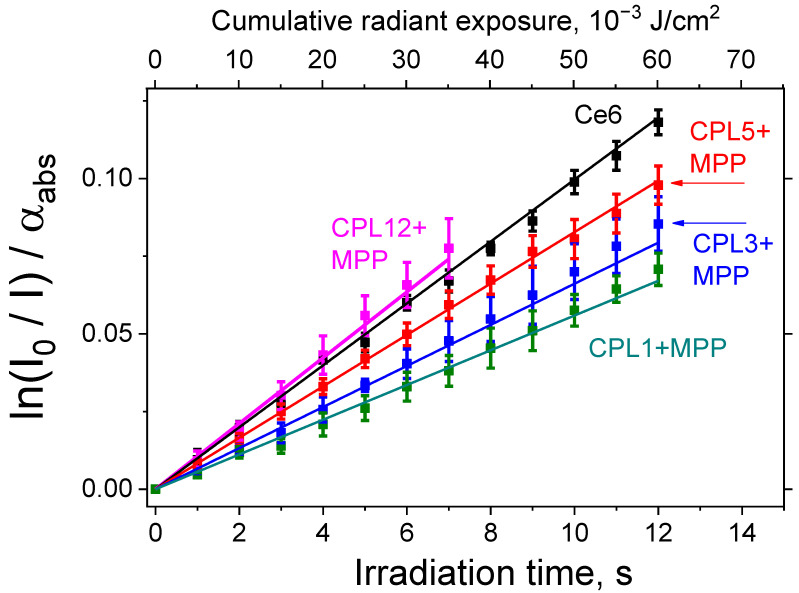
Kinetics of singlet oxygen generation, sensitized by nanoparticles under study or reference PS (Ce6) in water solution of lecithin liposomes with DPBF (^1^O_2_ probe) under red light irradiation (λ = 660 nm).

The MPP dye encapsulated in all NPs is also capable of effective ^1^O_2_ generation, however, for CPL1 + MPP, CPL3 + MPP, and CPL5 + MPP, the NPs’ Φ_Δ_ value is noticeably (1.5–2 times) lower than that for the reference dye Ce6, and only CPL12 + MPP generate ^1^O_2_ slightly efficiently (Figure 4 and Table 2). The observed difference for CPL12 + MPP and CPL1 + MPP NPs could be related to the CPL12 terpolymer structure—it contains hydrophobic units of cyclohexyl methacrylate, which allow MPP generate singlet oxygen more efficiently, compared to CPL1 copolymer, which consist only of VP and TEGDM units. The ^1^O_2_ generation efficiency of MPP encapsulated in CPL3 and CPL5 copolymers is slightly different, which could be explained by the presence of DT residues in CPL3 polymer chains and its influence on the dye.

### 3.6. Phototoxic Activity of Nanoparticles in HeLa Cancer Cells

The photodynamic activity of NPs aqueous solutions and water-soluble reference PS Ce6 was studied in the HeLa cell line. As can be observed from Supplementary Figure S6a, exposure to light (λ > 630 nm) for 30 min does not significantly affect the viability of HeLa cells under current experimental conditions.

We also evaluated the photo- and cytotoxic activity of the native copolymers. It was found that all copolymers, except for CPL3, are non-toxic at concentrations up to 10 mg/mL, which corresponds to an NPs concentration of ~100 µM (Supplementary Figure S6b,c). The pronounced cytotoxicity of CPL3 copolymer could be related to the presence of 1-decanethiol residues in its polymer chains (–SC_10_H_21_ groups, to be exact), as well as small amounts of DT itself.

**Figure 5:**
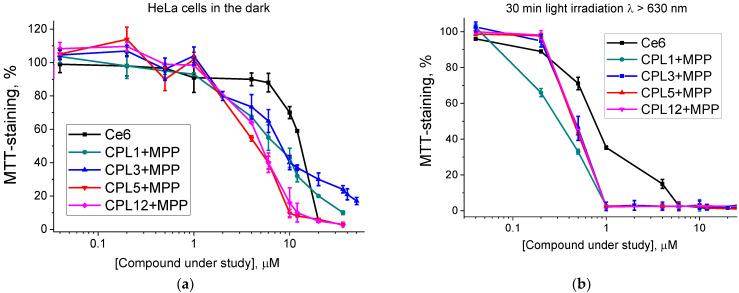
Phototoxicity of nanoparticles under study or reference PS (Ce6) in HeLa cancer cells: cells kept in the dark (**a**) and cells after 30 min light irradiation λ > 630 nm (**b**).

According to the results of the dark toxicity test, all NPs with MPP have either a comparable (IC_50_ = 12.1 μM for CPL3 + MPP) or 2–2.5 times higher cytotoxicity values (IC_50_ = 4.9 μM for CPL5 + MPP and 5.2 μM for CPL12 + MPP) than that for reference Ce6 (IC_50_ = 13.5 µM) (Table 2 and Figure 5a). We believe that it could be related to MPP’s much more pronounced hydrophobic properties compared to Ce6.

All NPs under study have a pronounced phototoxic effect (Figure 5b), and the IC_50_ dose of all NPs with MPP is 1.5–2 times higher than that for the reference dye Ce6 (IC_50_ = 0.83 μM, Table 2), which is one of the most effective PS currently used in clinical practice.

It should be noted that the cell medium with compounds under study in all experiments was removed and replaced with the fresh medium before the irradiation. Thereby, the photodynamic effect could be exerted only by MPP molecules, which either accumulated in the cells or were embedded in their membranes. Thus, the observed phototoxic effect of all the NPs under study could be considered an MPP ability to transfer from NPs into cells. Considering this fact, we decided to additionally analyze the interaction of NPs with model biological structures—liposomes and subcellular tissue homogenate.

### 3.7. Interaction of Nanoparticles with Liposomes and Tissue Homogenate

**Figure 6:**
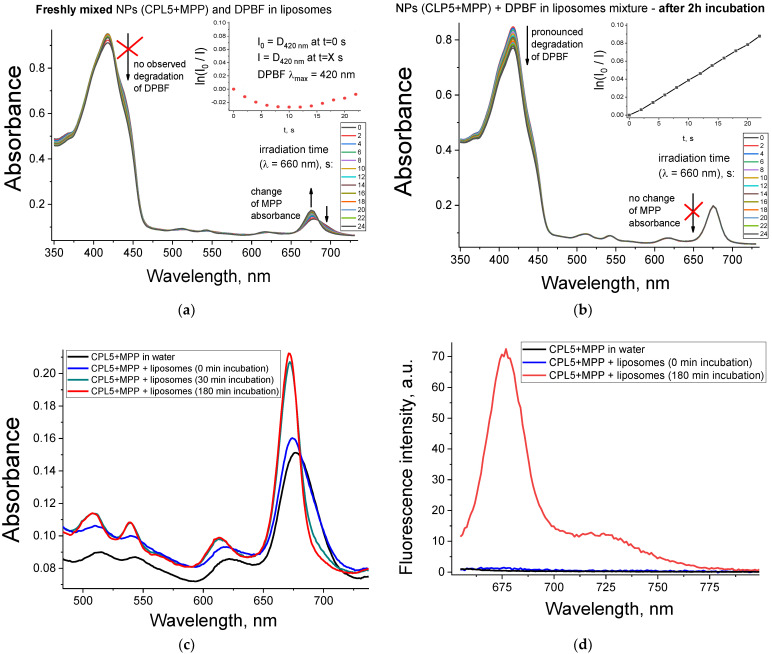
Interaction of CPL5 + MPP NPs with liposomes: absorption spectra of mixture for detection of singlet oxygen generation (DPBF probe encapsulated in lecithin liposomes)—freshly prepared (**a**) and after 2 h of incubation (**b**). The mixture of water solution of CPL5 + MPP immediately after addition to the lecithin liposomes solution (0 min) and after a certain incubation time (displayed in the legend)—absorption (**c**) and fluorescence spectra (**d**).

In addition to the results in HeLa cells, during the analysis of the singlet oxygen generation, we found another evidence of the ability of MPP transfer from NPs to membranes. If a mixture of NPs water solution and DPBF probe in lecithin liposomes is irradiated immediately after its preparation, we observe a pronounced change in the absorbance of the Q-band region, while almost no photodegradation of the DPBF probe is observed (Figure 6a). However, if the same mixture is incubated for some time (2 h) in the dark at room temperature, then the picture drastically changes: the absorption of the MPP remains the same, and the DPBF label is degraded much more efficiently (Figure 6b).

In Figure 6a,b, this effect is illustrated by the example of CPL5 + MPP; however, it is also observed for all the studied NPs. We assume that the observed effect is associated with the ability of MPP to gradually transfer from polymeric NPs to the membranes of lecithin liposomes, in which the DPBF probe is located. If light irradiation is performed immediately after mixing the NPs and DPBF solutions, then the MPP does not have time to transfer from NPs into liposomes. However, if the MPP has enough time to transfer from NPs to liposomes, then we observe MPP a in much less aggregated form, as could be concluded from the absorbance of the MPP Q-band region. The observed effect is well-known in the literature—many works discuss the release of the active compound from amphiphilic NPs into cell membranes [15]. This effect is also used for the creation of liposomes loaded with a hydrophobic compound (native C_60_ fullerene) due to its transition from amphiphilic NPs to “empty” liposomes in an aqueous solution [49].

Reference compound Ce6 also can interact with liposomes—we observe a pronounced change in the absorbance of the Q-band region of Ce6 when we mix it with a water solution with liposomes (Supplementary Figure S5a). Still, in that case, it happens almost instantly, contrary to a much slower process for MPP-loaded NPs under study. We believe that Ce6 transfer from the aggregated form in water solution to the non-aggregated form in liposome membranes due to its amphiphilic properties—Ce6 has a large hydrophobic macrocycle ring and three polar acid groups (Figure 1b).

Disaggregation of MPP upon transfer from NPs to membranes has two crucial effects for application in PDT. First, it significantly increases the singlet oxygen generation ability of MPP (Figure 6b). It is in good agreement with the literature data, since the aggregation of PS significantly reduces its ^1^O_2_ quantum yield [50].

Second, disaggregation of MPP should increase the fluorescence signal from MPP. To confirm that, we mixed a water solution of CPL5 + MPP with a solution of “empty” liposomes (without a DPBF probe). The MPP in the obtained mixture demonstrated a gradual change of absorbance in the Q-band region (Figure 6c) and a sharp increase (by ~50 times) of fluorescence signal after incubation for 3 h (Figure 6d).

To test the assumption that MPP could transfer from the NPs not only to the membranes of lecithin liposomes but also to other biological structures, we analyzed the fluorescence spectra of the NPs in a solution of a tissue homogenate.

As can be observed from Supplementary Figure S7, an incubation of NPs with highly diluted subcellular mouse brain homogenate (protein concentration 0.01 mg/mL) leads to a gradual increase in the fluorescence signal of MPP for all NPs under study. We believe that MPP transfer from NPs to hydrophobic components of the tissue homogenate—cells membranes and protein hydrophobic sites. Most part of MPP transferred from NPs to biological structures within the first 15 min of incubation and reached its peak by one hour of incubation for all studied NPs, except for CPL5 + MPP—these NPs gradually released MPP during all 2 h of incubation (Supplementary Figure S5c). The slower release of MPP from NPs based on CPL5 could be related to its polymer chains’ higher molecular packing density compared to other copolymers.

Thus, the observed effect of the gradual release of PS from studied NPs is important not only for achieving higher phototoxicity of PS, but also for application in fluorescent diagnostics. It can be expected that the PS, encapsulated in the NPs, will retain in the aggregated (non-fluorescent) form in a water solution. At the same time, during accumulation in the tumor cells of the PS, its aggregation degree significantly reduces and sharply increases its fluorescence signal. This effect will achieve a higher signal-to-noise ratio and a clearer tumor image in fluorescence diagnostics.

### 3.8. Storage Stability of the Copolymer + MPP Nanoparticles

Stability after long-time storage (i.e., “shelf life”) is one of the main requirements for a drug candidate. Drugs have to retain their properties after a long period of storage, which could vary from 6 to 36 months, depending on the regulation protocol used in the current country [51].

**Figure 7:**
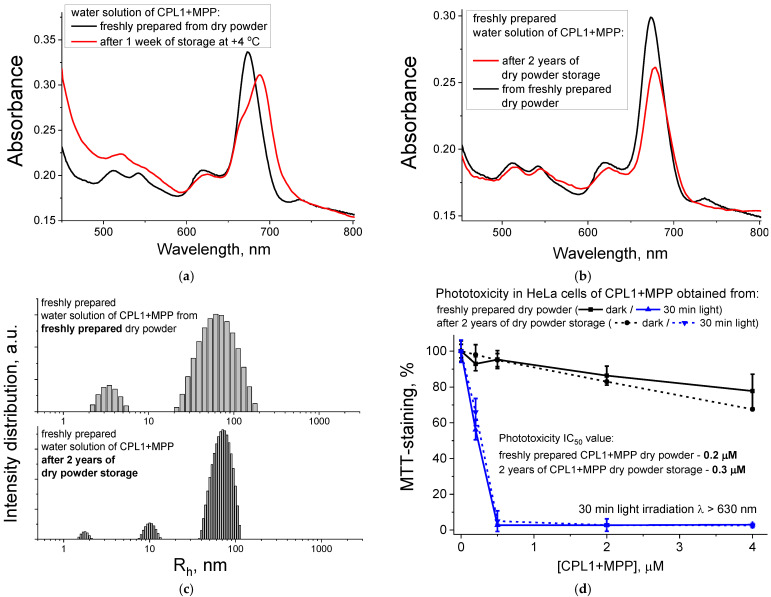
Properties of CPL1 + MPP freshly prepared and after storage time—absorption spectra after 1 week of solution storage at +4 °C (**a**) and a comparison of freshly prepared water solution from just obtained dry powder and powder stored for 2 years: absorption spectra, same MPP concentration (**b**), DLS profiles of NPs sizes, 20 °C (**c**), and phototoxic action (**d**).

The stability of water solutions of nanoparticles is a well-known problem: they tend to aggregate and lose their properties within several days of storage [52]. As observed in Figure 7a, after one week of CPL1 + MPP water solution storage at +4 °C, we observe a change in the absorbance of the Q-band region of MPP, which indicates the facilitation of MPP aggregation and formation of NPs gel-like precipitate on the bottom of the solution. The same results were for the PBS solutions. One of the generally accepted ways to solve this problem and increase the shelf life of NPs is the preparation of lyophilized dry powder [52].

Since the applied in the current work method of NPs preparation already included the stage of dry powder (Section 2.3 and Supplementary Figure S2c–f), we compared the properties of aqueous solutions of CPL1 + MPP NPs prepared from two types of dry powder: freshly obtained one and another, which was stored for two years at +4 °C in the dark. We observe almost no change in the absorbance spectra, except the slight decrease (by 1.2 times) of the absorbance of the Q-band region of MPP for the old dry powder compared to the new one (Figure 7b). No significant change was observed for DLS size distributions of NPs—their R_h_ value remained around 77 nm for most NPs (Figure 7c). Furthermore, the phototoxic activity of the CPL1 + MPP NPs in HeLa cells practically does not change even after 2 years of storage—the IC_50_ phototoxicity value slightly changes from 0.2 to 0.3 μM, while they retain the same dark toxicity level for the MPP concentrations up to 4 μM (Figure 7d).

As can be concluded from the above-listed results, when stored as a dry powder, NPs under study can retain their properties for a long time (up to two years), as shown in the example of CPL1 + MPP NPs, which is extremely important for the future development of potential nanoparticle-based drugs.

## 4. Conclusions

Thus, in the current work, we obtained four different compositions in the form of dry powder, in which hydrophobic dye methylpheophorbide *a* was encapsulated in amphiphilic copolymers of N-vinipyrrolidone. When dissolved in PBS water solution, these compositions form nanoparticles ranging in hydrodynamic radius from 20 to 100 nm. As it was concluded from absorption spectra and highly quenched fluorescence signal, the dye embedded in these nanoparticles is in an aggregated state.

The obtained nanoparticles can efficiently generate singlet oxygen when irradiated with red light (λ = 660 nm), as was shown in a model system based on lecithin liposomes and a specific probe of 1,3-diphenylisobenzofuran. The singlet oxygen quantum yield of the obtained NPs is comparable to that for the water-soluble reference photosensitizer, trisodium salt of chlorin e_6_. The in vitro photodynamic activity of the obtained nanoparticles, estimated in a HeLa cell culture, exceeds 1.5–2 times that of the reference photosensitizer, which is currently one of the most effective photosensitizers used in clinical practice.

Despite high aggregation of the dye in the water solution of nanoparticles, upon the interaction of these NPs with model biological membranes—liposomes or components of a tissue homogenate, the dye aggregation degree decreases sharply, and singlet oxygen quantum yield and the fluorescence signal significantly increase due to the gradual release of the dye from the nanoparticles.

It has been demonstrated that the obtained (copolymer + dye) compositions in the form of dry powder can be stored for a long time (two years or more), and when they are dissolved in water, they have retained the same absorbance spectra, nanoparticle size, and high phototoxicity level.

Thus, the approach used in this work allows obtaining (copolymer + dye) nanoparticles that have high water solubility, stability, and a pronounced photodynamic effect, which show promising potential of such nanoparticles as a modern platform for the design of targeted delivery vehicles for photodynamic therapy, fluorescence diagnostics, and other biomedical applications.

## Figures and Tables

**Table 1:** Physicochemical characteristics of the VP copolymers.

The Copolymers	The Content of VP and (di)Methacry-Lates Units in Copolymers, mol%	*M*_w_, kDa	CAC, mg mL^−1^	*R*_h_, nm
CPL1	94.6:5.4	24.0	4.1	4; 66
CPL3	86.5:9.9	19.0	1.7	4; 77
CPL5	90.7:9.3	120.0	3.6	2; 31
CPL12	90.9:9.1	126.0	0.55	50

**Table 2:** A comparison of photophysical properties and photodynamic activity of reference PS and nanoparticles under study.

	MPP	Ce6	CPL1 + MPP	CPL3 + MPP	CPL5 + MPP	CPL12 + MPP
Solvent	Toluene	PBS	PBS	PBS	PBS	PBS
Range of DLS hydrodynamic sizes *R*_h_, nm	−	27 ± 20	77 ± 25	31 ± 15	95 ± 25	57 ± 10
Q-band λ_max_, nm	669	647	675	687	675	675
Fluorescence λ_max_, nm	677	653	671	672	676	676
Relative fluorescence quantum yield	1	1/7	1/249	1/108	1/258	1/65
Relative singlet oxygen quantum yield Φ_Δ_, %	−	0.75	0.4	0.5	0.63	0.8
Dark cytotoxicity in HeLa cells (IC_50_), μM	−	13 ± 1.1	5 ± 0.4	12.1 ± 0.6	5.2 ± 0.9	4.9 ± 0.2
Phototoxicity in HeLa cells (IC_50_), μM	−	0.83 ± 0.13	0.31 ± 0.03	0.5 ± 0.02	0.4 ± 0.07	0.3 ± 0.05

## Data Availability

Not applicable.

## Supplementary Materials

The following supporting information can be downloaded at: https://www.mdpi.com/article/10.3390/pharmaceutics15010273/s1, Figure S1: Synthesis of the chlorin e_6_ water-soluble salt (Ce6) according to the method described in ref. [25,26] from the article; Figure S2: Topological structures of VP−TEGDM (CPL1) (a) and VP−PEGMEM−TEGDM (CPL3 or CPL5) and VP−CHM−TEGDM (CPL12) (b) copolymers. Photos of dry powders and water solutions (50 μM) of obtained nanoparticles: CPL1 + MPP (c), CPL3 + MPP (d), CPL5 + MPP (e), CPL12 + MPP (f); Figure S3: FTIR spectra of CPL1-CPL12, MFF and their copolymer compositions; Figure S4: The intensity distribution of light scattering over the size of scattering centers of CPL12 in water solution. [CPL12] = 0.62 mg/mL; Figure S5: Absorbance (a) and fluorescence (b) spectra of native MPP (2 μM), reference PS Ce6 (2 μM) in water solution, solution of lecithin liposomes and DMSO. For all fluorescence spectra λex = 420 nm; Figure S6: Phototoxicity of negative control (cells without PS, a), water solutions of native copolymers after 30 min light irradiation (b) and kept in the dark (c) tested in HeLa cells; Figure S7: Fluorescence spectra of an aqueous solution of CPL1 + MPP (a), CPL3 + MPP (b), CPL5 + MPP (c), CPL12 + MPP (d) with the addition an aqueous solution of mouse brain subcellular homogenate (protein concentration 0.01 mg/mL) immediately after addition of NPs (0 min) and after certain incubation time (displayed in the legend) at room temperature. For all fluorescence spectra λex = 640 nm.
