# Supplementary material for: Nanoparticles of N-Vinylpyrrolidone Amphiphilic Copolymers and Pheophorbide a as Promising Photosensitizers for Photodynamic Therapy: Design, Properties and In Vitro Phototoxic Activity
Source: Pharmaceutics. 2023 Jan 12;15(1):273. doi: 10.3390/pharmaceutics15010273 (PMC9863766; doi:10.3390/pharmaceutics15010273)
Supplement: Supplementary file 1 [file pharmaceutics-15-00273-s001.zip › pharmaceutics-2121180-supplementary.pdf]

pharmaceutics

MDPI

Supplementary Materials: Nanoparticles of *N*-Vinylpyrrolidone Amphiphilic Copolymers and Pheophorbide a as Promising Photosensitizers for Photodynamic Therapy: Design, Properties and In Vitro Phototoxic Activity

Alexander Yu. Rybkin, Svetlana V. Kurmaz, Elizaveta A. Urakova, Natalia V. Filatova, Lev R. Sizov, Alexey V. Kozlov, Mikhail O. Koifman and Nikolai S. Goryachev

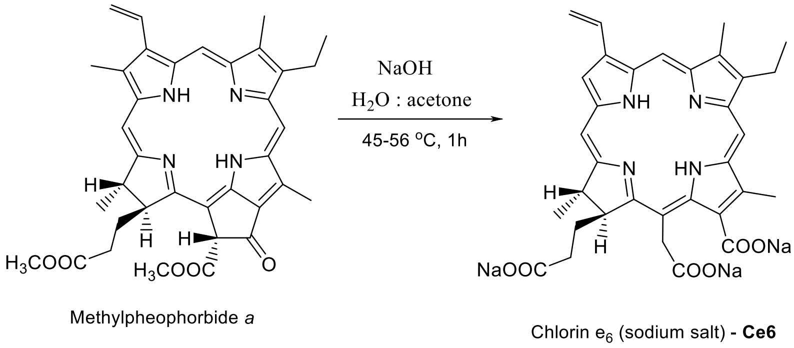

Figure S1. Synthesis of the chlorin e6 water-soluble salt (Ce6) according to the method described in ref. [25,26] from the article.

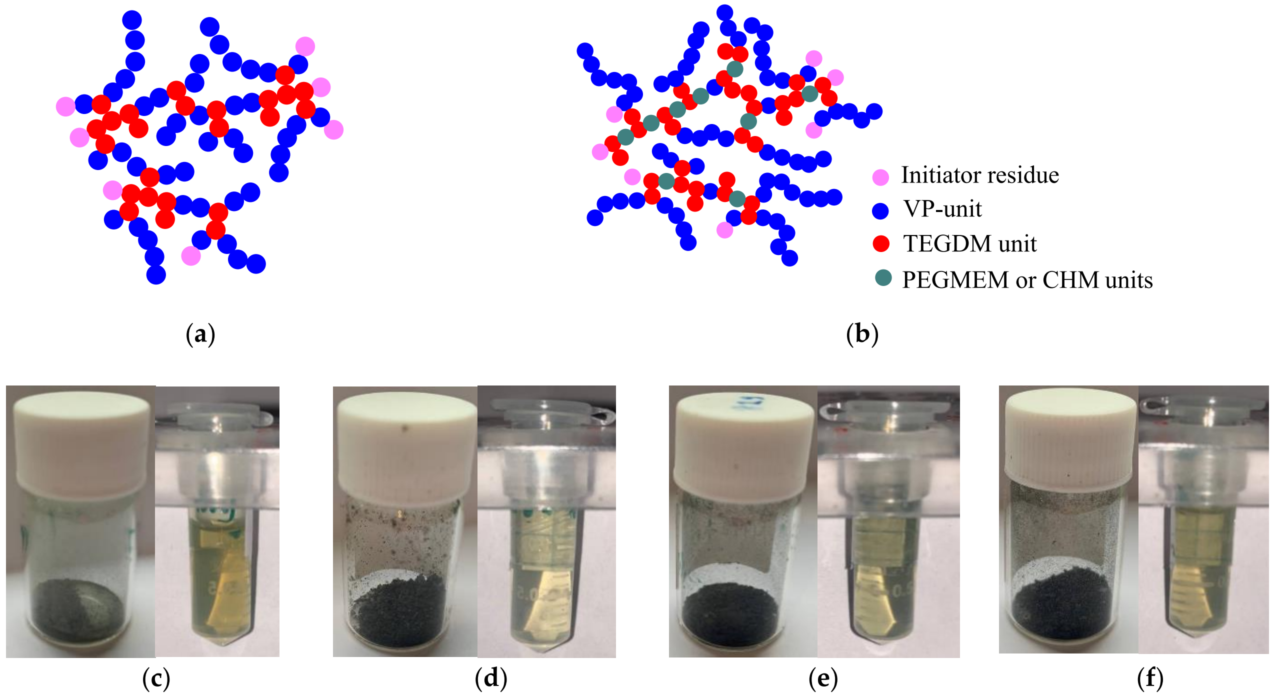

Figure S2. Topological structures of VP−TEGDM (CPL1) (**a**) and VP−PEGMEM−TEGDM (CPL3 or CPL5) and VP−CHM−TEGDM (CPL12) (**b**) copolymers. Photos of dry powders and water solutions (50 μM) of obtained nanoparticles: CPL1+MPP (**c**), CPL3+MPP (**d**), CPL5+MPP (**e**), CPL12+MPP (**f**).

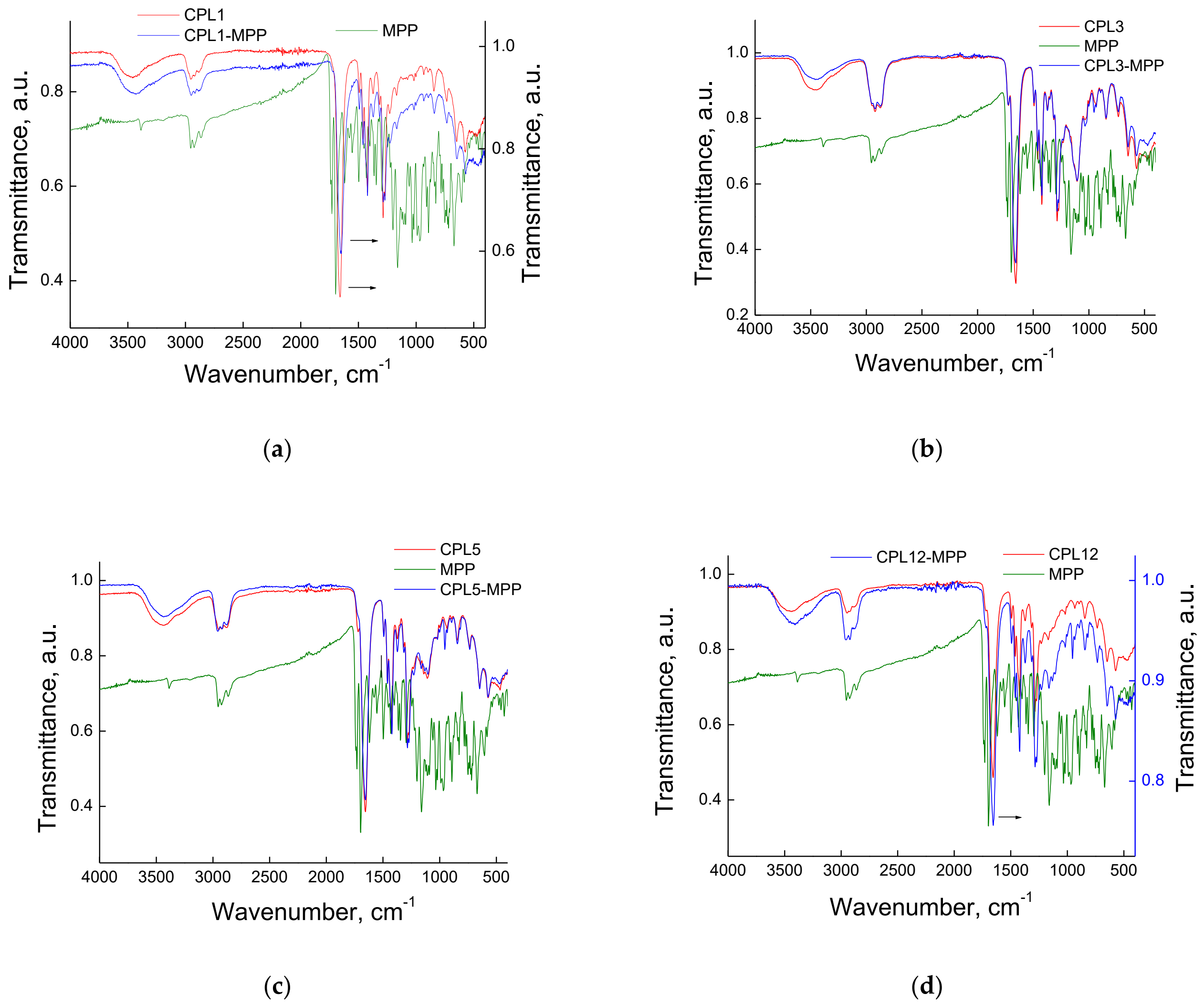

Figure S3. FTIR spectra of CPL1-CPL12, MFF and their copolymer compositions.

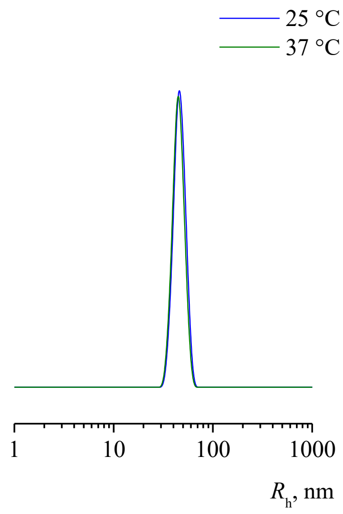

Figure S4. The intensity distribution of light scattering over the size of scattering centers of CPL12 in water solution. [CPL12] = 0.62 mg/mL.

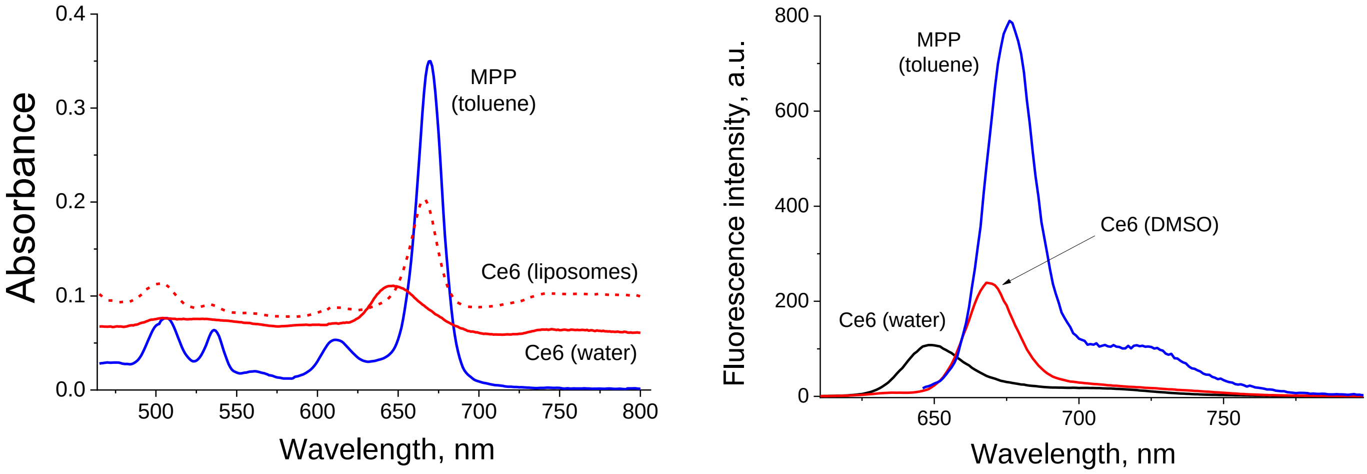

(**a**) (**b**)

Figure S5. Absorbance (**a**) and fluorescence (**b**) spectra of native MPP (2 μM), reference PS Ce6 (2 μM) in water solution, solution of lecithin liposomes and DMSO. For all fluorescence spectra λ_{ex} = 420 nm.

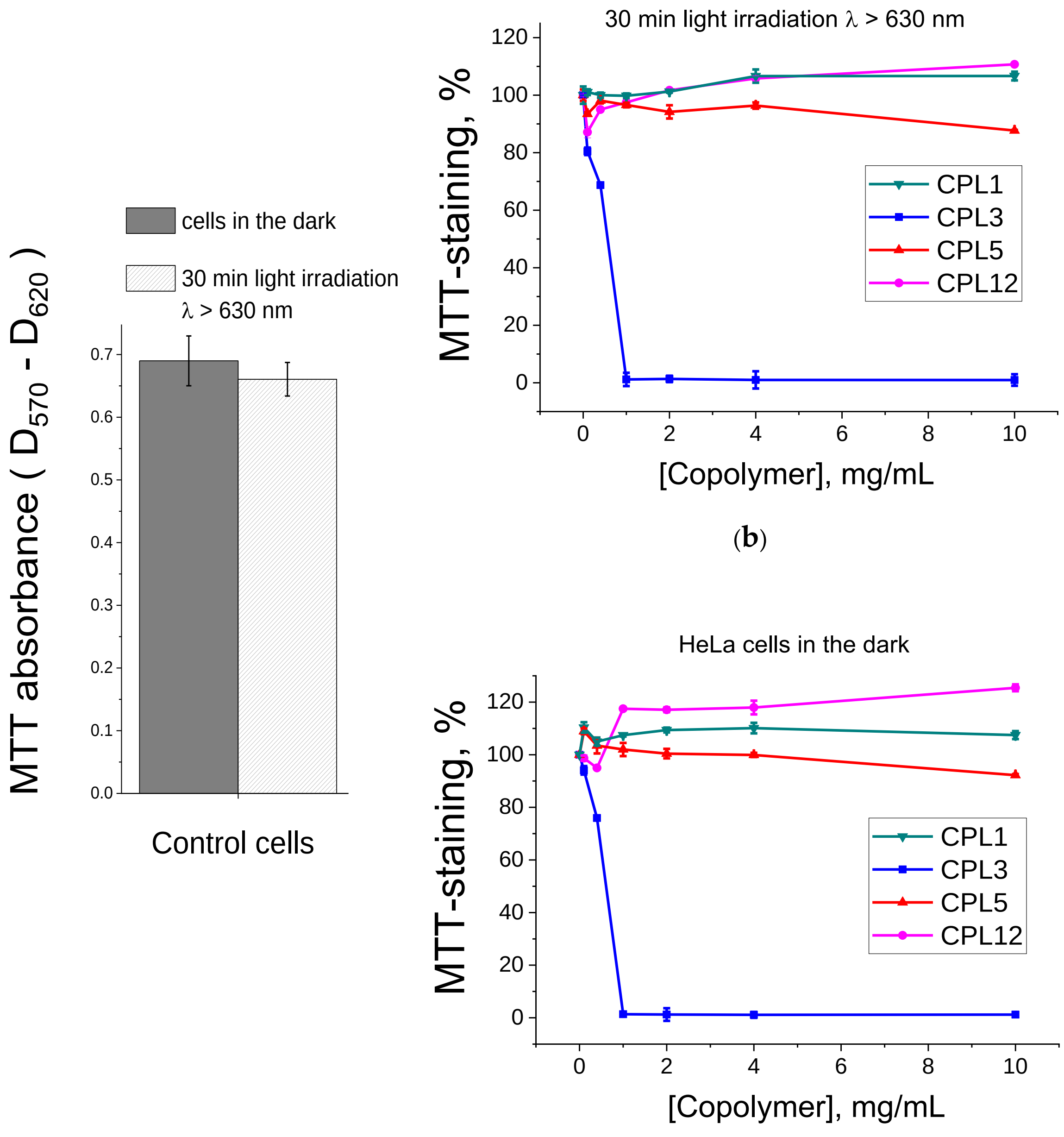

(**b**)

(**a**) (**c**)

Figure S6. Phototoxicity of negative control (cells without PS, **a**), water solutions of native copolymers after 30 min light irradiation (**b**) and kept in the dark (**c**) tested in HeLa cells.

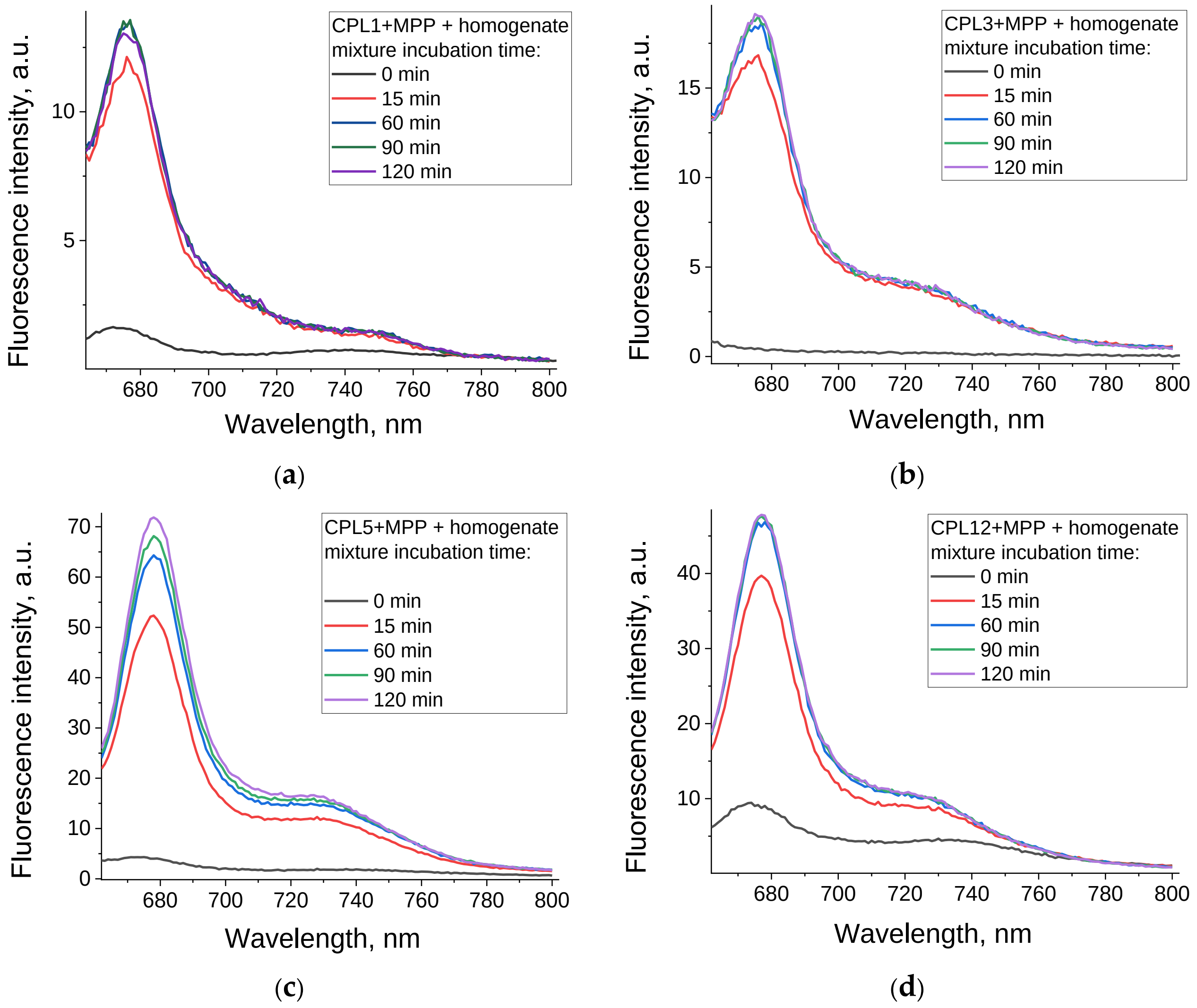

Figure S7. Fluorescence spectra of an aqueous solution of CPL1+MPP (**a**), CPL3+MPP (**b**), CPL5+MPP (**c**), CPL12+MPP (**d**) with the addition an aqueous solution of mouse brain subcellular homogenate (protein concentration 0.01 mg/mL) immediately after addition of NPs (0 min) and after certain incubation time (displayed in the legend) at room temperature. For all fluorescence spectra λ_{ex} = 640 nm.
